# Surgery

**Published:** 1877-02

**Authors:** 


### II. SURGERY.

1. *A New Method of Examining the Affections of the Vocal Cords.* KLEMM. (*Arch. d. Heilk*, No. 6, 1876.)

The readiness with which a small flame is set into motions by the vibrations of the air has been utilized by Dr. Klemm for taking photographs, as it were, of the normal and abnormal vibrations of the vocal cords. His apparatus consists of, 1, a large cube rotating on a perpendicular axis, with its four vertical sides lined with glass mirrors; 2, a gas burner terminating in a fine point, so as to give a very small pointed light. To this burner are attached a wide speaking tube of rubber and another rubber tube which furnishes the gas. The speaking tube and gas tube are separated by a very delicate membrane of caoutchouc. Any tone spoken or sung into the speaking tube sets this thin membrane in vibrations, which are communicated to the gas and the light; the light thus responds to the feeblest vibrations of that membrane. The cube is placed about eighteen inches in front of the light, and the room is darkened. If the cube then is made to revolve with moderate speed on its perpendicular axis, the quiet light reflected by the glass mirrors gives the observer the impression of a continuous light streak; but if the light is moved by the vibrations of a tone sent through the speaking tube, flashes shoot up from the light streak in regular intervals. These flashes have a very regular shape, like the long sharp teeth of a saw, and are separated by regular narrow, but deep, indentations. The shape and height of these light teeth depend, 1, on the pitch of the tone uttered; 2, on its power; 3, on the vowel uttered; 4, on the quality of the voice. As the first three conditions can easily be controlled and made uniform for a series of observations, we can from the images of the light teeth draw certain conclusions concerning the vibrations of the vocal cords.

In a series of drawings executed by a skillful artist, the salient features are illustrated in the images produced by normal and abnormal vibrations of the vocal cords. The high tones of the musical scale produce single long teeth; by middle tones these teeth are split in two, each pair of teeth being separated by a deep indentation from the next one, and the lower tones are characterized by groups of three or four teeth; but all these teeth ending in a sharp distinct point. By a moderate degree of hoarseness the points become blunted, the teeth broader and shorter, and the indentations shallower.

The pictures of very hoarse voices show a great irregularity of the teeth in length and width, so that the light streak resembles a row of fringes; and in a case of excessive hoarseness (as due to paralysis of the vocal cords) the teeth become so short and indistinct that the whole streak looks more like a scalloped ribbon.

2. *New Method of Curing Phymosis.* GRIFFITH. (*British Med. Journal*, No. 823.)

Three cases of phymosis are reported in which the surgeon did not perform any of the usual operations, but enlarged the aperture in the prepuce by distention. This dilatation may be effected rapidly, in one sitting, especially if we do not mind splitting the mucous lining of the foreskin, or it is accomplished gradually in two and more sittings. In the first case the patient had a very slight stricture of the prepuce, which was effectually dilated by means of a forceps something like the ordinary uterine dressing forceps. The pain was very trifling. In the two other cases the patients had never been able to uncover any part of the glans except the very mouth of the urethra. These cases were cured by gradual dilatation, which the doctor describes thus: "I grasped the penis with the fingers of both hands, and partly by retracting the foreskin, partly by projecting forward the glans penis, I commenced this wedge-like dilatation, and soon had the satisfaction of seeing an area of surface around the mouth of the urethra." This procedure was repeated every other day until the phymosis was cured so as not to be likely to return. An injection of some oil under the foreskin, previous to each sitting, seems to facilitate the dilatation and to shorten the whole treatment.

3. *Boracic Acid as an Ordinary Dressing for Wounds.* CANE. (*London Lancet*, Aug., 1876.)

The writer calls attention to the use of boracic acid as a *simple* dressing, altogether apart from the antiseptic system, for all kinds of wounds. While the advantages of Mr. Lister's method are acknowedged, Dr. Cane claims that we want something which, while it has to a certain extent the merits of Lister's method, is without those tedious details, and which can be readily used without assistance.

After an extensive trial of the acid for some months, the author has secured better results than from the ordinary methods of dressing. The most convenient forms for use are the boracic lint, cotton wool, a concentrated watery solution of acid, and boracic ointment.

Boracic lint is prepared by soaking lint in a saturated *boiling* solution of the acid. Upon drying the lint a deposit of fine, flaky crystals takes place between its fibres. Cotton wool is similarly prepared, and when dried and picked out forms a very useful dressing. The concentrated solution is made by dissolving the acid in boiling water to saturation. The ointment is made by rubbing down one drachm of the acid with one ounce of simple ointment or benzoated lard. Boracic acid, unlike most antiseptic agents, is blank and unirritating, but being non-volatile it is less useful in some cases than carbolic acid. The author recommends the boracic lint to be used in a dry state. I have, however, seen it used in a wet state by Prof. Gunn, of this city, in the case of a compound injury of the ankle joint, with the most gratifying result.

Dr. Cane has found the acid very useful in skin grafting. The surface to which the grafts are to be applied is first well cleansed by applying the boracic lotion some days, till the discharge is free from putrefactive ordor. The skin from which it is intended to take the grafts is also well washed with the lotion. The grafts are applied to the raw surface in the usual way, after which they are protected by strips of gutta percha tissue dipped in the boracic lotion, and a piece of boracic lint placed over all.

4. *A Simple Mode of Extracting Calculi Arrested in the Urethra.* WILL. (*London Lancet*, Aug., 1876.)

Dr. Will succeeded, in two cases, by means of a silver wire, in removing calculi arrested in the urethra. One case was complicated with stricture. A loop of tolerably thick silver wire was passed through the stricture and behind the calculus. After some manipulations the latter became engaged in the wire loop, and although slipping from the loop more than once, was finally withdrawn. The second case was that of a child, the calculus having lodged in the bulb of the urethra.

5. *Removal of a Button from the Bronchus.* (*London Lancet*, Aug., 1876.)

The patient, aged 13 years, accidentally slipped the button into his trachea, April 23d, where it remained without producing very serious symptoms until May 11th, when it fell into the left bronchus, producing symptoms of collapse of the lower lobe of the lung. Mr. Maundee having performed tracheotomy, first inverted and shook the patient, but without success. He then placed the patient on his back and pressed through the wound into the left bronchus about seven inches of looped silver wire, and was successful in withdrawing the button, together with a quantity of muco-purulent matter. The urgent symptons disappeared.

6. *Embolism of the Pulmonary Arteries in Consequence of the Application of Elastic Bandages to the Lower Extremities.* MASSARI. (*The Boston Med. and Surg. Jour.*, June 29, 1876.)

* The bandages were applied after confinement in which the patient, in consequence of placenta prævia, had become extremely anæmic. The symptoms of anæmia disappeared remarkably quickly and returned again immediately, when, in consequence of violent pain, the bandages were twice loosened, for a short time. Their third removal, thirty-two hours after delivery, was followed by sudden collapse, dyspnea, violent heart beats, and death in two hours, in spite of a re-application of the bandage s Clots were found in the pulmonary arteries of the third order, and similar ones in the varicose saphena veins. The author regards the bandaging as the cause of the coagulation, and considers its employment contra-indicated by a varicose condition of the veins. He also gives a caution against the too prolonged application of the bandages.

7. *Extirpation of the Parotid Gland by the Galvano-Cautery.* (*Med. and Surg. Reporter*, Sept. 2, 1876.) [Translated by Jno. B. Roberts, M.D., of Philadelphia.]

The patient, a man aged 58 years, had had, for nine months, lancinating pains in the left ear, radiating over the whole side of the head. There was some paralysis of the face. When the patient opened the mouth he became aware of a mechanical impediment between the jaw and the ear. Prof. Carradi diagnosed a tumor of the parotid gland, either carcinoma or enchondroma, compressing the left facial nerve, and decided upon extirpation of the gland. In order to avoid hæmorrhage and have the field of the operation clear, the galvano-cautery was employed. A vertical incision was made, extending from the zygoma to a point below the angle of the jaw, being almost half an inch in front of the attachment of the external ear; a second incision started from the lower angle of the masseter muscle and reached the mastoid process. These cutaneous wounds having been dried, a steel knife, made white hot by the galvanic current, was employed and the whole gland was separated from the surrounding tissues from below upward. During the last part of the dissection, the external

* Translation.

carotid having been exposed, two ligatures were applied and the vessel divided.

At the end of 20 days the patient was well, but the paralysis of the left eye and side of the face persisted. Subsequently a swelling of the left lateral region of the neck appeared, showing a recurrence of the disease, which the microscope had proved to be scirrhus.

8. *A new View of the Pathology of the so-called Phlebitis.* Nancude. (*Philadelphia Medical Times*, Aug. 19, 1876.)

As to the pathology of so-called phlebitis, this paper supports the theory that thrombosis is the primary affection, the inflammation being secondary in the order of occurrence. The author, as well as some of the modern pathologists, denies the existence of adhesive inflammation of the veins, as indicated by an effusion of plastic lymph upon the intima, causing a secondary thrombosis. The experiments of Gendrin long obtained credit and gave support to the doctrine of adhesive phlebitis till Lee and Callender proved their inaccuracy. Frances showed by specimens that veins after ligature or division repaired without adhesive inflammation.

It is difficult to conceive of a non-vascular tissue, such as the intima, initiating inflammatory changes. Then, too, some lymph effused, it is not easy to understand how it could remain in its soft semi-fluid condition attached to the vascular walls long enough to form false membrane, or by its roughness to indicate coagulation of the blood. On such grounds the author feels compelled to strike out the term phlebitis and substitute that of thrombosis.

Dr. Nancude's "New View of the Pathology of Phlebitis" is based upon the demonstrations of Alex. Schmidt, to the effect that fibrine, as such, does not exist in the blood, but when a clot forms it is due to the union of two substances always present in the blood, namely, fibrino-plastic and fibrinogen. The former is present chiefly in the blood cells, and the latter is the result of the retrograde metamorphosis of tissue, and is found in the serum. Before these observations were published there was no satisfactory explanation of the so-called hypermosis, so constant an attendant in rheumatic fever, traumatism, pneumonitis, and in the puerperal state. Increased tissue waste with inadequate action of the secerning organs result in an immense excess of the fibrino-genetic material. Particularly is this the case in the puerperal state, where we too frequently see thrombosis, and where an enormous mass of uterine tissue is absorbed in the short space of six weeks, the greater part of this absorption taking place during the first ten days. Under these circumstances it must be evident to what degree the blood is surcharged with fibrine-forming substance.

The views usually entertained concerning the immediate cause of the thrombosis really amount to nothing, for they refer us to some unknown condition of the system. The author claims that his own views of its causation render it perfectly plain. In all cases of this affection the wound is eminently unhealthy, and it is therefore in a condition that favors the absorption of the products of retrograde metamorphosis; that is one factor. The other is that in natural hæmostasis, after any lesion of continuity, we have a clot sealing the opened ends of the vessels. When the retrograde products reach the plugged vein by the next uninjured collateral, they meet here the fibrino-plastic substance in the hæmostatic plug, and we have a thrombus formed, extending toward the heart. The next collateral above brings in fresh fibrino-genetic matter which meets the thrombus and it again extends. The clotting ceases only when no more collateral veins pour in blood coming directly from the disease focus, and therefore loaded with fibrino-genetic matter.

*Post mortem* reports* furnish evidence which sustains the views herein expressed, the clot always ceasing when no more impure blood is poured directly into the affected veins, rather than where the main vein joins the diseased one according to most writers. The paper concludes with a consideration of the practical bearing of this new pathological view on prophylaxis and treatment, viz.: To remove or render inert all dead or dying tissues and inflammatory products, and avoid caustic and irritating applications likely to chemically or mechanically injure the granulations; thus rendering them capable of absorbing.

Mr. Lister and his adherents think that the excellent results of the modern management of wounds are owing to the destruction of microscopic organisms; Dr. Nancude to the rendering inert retrograded substances, and to the prevention of such retrogression, in conjunction with the avoidance of any injury whatever to the granulations.

9. *Fracture of Cranium Dating Back* 30 *Years; Persistent Fistula; Integrity of Cerebral Function for* 30 *Years; Signs of Compression; Trephining and Cure.* CRAS. (*L'Union Médicale*, No. 153.)

Last October the reporter was summoned to a patient in a state of profound coma, with purple features, relaxed limbs, stertorous respiration and pulse between 120 and 130. The forehead was evidently protruding and was the site of a cicatrix traversed by a fistula. The patient's wife informed the surgeon that the cause of this fistula was a fall which the husband had many years before. This had produced a wound which had completely healed, but had always discharged a little matter. For four or five years afterward the husband had suffered greatly; his intellectual faculties, formerly unimpaired, became weakened. She had noticed the occurrence of crises which coincided with the cessation of the discharge from the frontal fistula. Cras applied Vienna paste and explored the fistula for several millimeters without encountering denuded bone.

At evening the incision of the eschar gave exit to an abundant discharge of viscid pus, under the influence of which the patient recovered consciousness and gave full details of his case, stating that the accident had occurred 38 years before. For five years he had been unable to labor. Suffering as he did from persistent cephalalgia with progressive amnesia,

* Holmes' System of Surgery, vol. iii., p. 361.

left hemiplegia and frequent accesses of fever. During the preceding 18 months his health had been worse than heretofore.

Cras decided that a large purulent collection was compressing the right hemisphere, and in spite of the amelioration already produced, and in the face of much opposition from the patient's family, insisted upon further operative procedure.

Local anæsthesia was induced by the employment of ice and sea-salt, when the operator (Jules Richard assisting) denuded the frontal bone to the entire extent of the fistula by means of an incision, involving all the superficial tissues and periosteum. The wound being dried, a large trephine was applied and made to penetrate slowly the thickened layers of the hypertrophied frontal bone. The instrument was succeded by the gouge and mallet, which were necessary to remove the superficial layers of the bony disk, when the trephine was re-applied, and at last its inferior segment had tapped the inferior wall of quite a large cavity, which gave exit to several drops of pus immediately. With a few more turns of the instrument the disk was loosened and the elevator, introduced at once, broke away a long bony plug. At the same time a stream of greenish pus bathed the face of the patient, the exit occurring by jets synchronous with the beating of the pulse. The quantity thus escaping measured 250 to 300 grammes.

The cavity was washed out with tar emulsion and the wound dressed with charpie, the patient exhibiting a remarkable return of intellection.

After some delays the case progressed with astonishing results. The intelligence was perfectly restored, the patient soon reading, writing and attending to his daily affairs. Meantime a small silver cannula was kept in the wound as a drainage tube, 1 mm. in diameter. One year afterward the patient still wore, by way of precaution, a small cannula, which was left open at night, and which contained only three or four grammes of a clear limpid fluid. The restoration of muscular vigor was proportioned to the cerebral energy.

"It was a true resurrection," concludes the author, "and when I saw him lately jump leisurely into a tilbury, and drive a spirited horse with one sure hand, I experienced a genuine satisfaction in thinking of the wretched and blinded imbecile transformed so happily by the intervention of surgery."

10. *Luxation of the Foot Backward.* Courbis. (*Lyon Médical*, No. 48.)

A young man who was engaged in play with older and stronger companions accidentally fell to the ground upon the back, while his left foot was engaged beneath the body of his adversary who also fell. The foot was thus forcibly extended.

When examined, immediately after the accident, little swelling had occurred. The dorsum of the foot was slightly shortened by reason of the propulsion forward of the tibio-fibular articulation. The anterior articular edge of the tibia projected to a marked degree, and had ridden over the astragalus, from which it was separated by a very distinct depression. The foot was at right angles to the leg, without deviation to either side.

The two malleoli displaced forward projected moderately, especially on the internal face of the member—the moderate swelling elsewhere masking this feature. The fibula was not fractured.

Posteriorly the injured limb offered a striking contrast to that which was unaffected. The heel seemed to be enlarged in consequence of the displacement forward of the malleoli. The tendo Achillis, which had partly followed the tibia, formed a curve, whose concavity was posterior, and was much more marked than on the opposite side. Tracing the course of each side upon a sheet of paper and describing a vertical line at right angles to the sole, is was found that the greatest distance between the arc and the chord of the sound side was 17 mm., while the greatest distance between the points on the side of the luxation was 39 mm.; showing that the tibio-peroneal displacement was at least to the extent of 24 to 25 mm.

The looseness of the union between the tendo Achillis and tibia shows that the real displacement of the bone is somewhat more considerable than is indicated by the tracings.

Movement of the part was restricted to slight flexion, and extension accompanied by intense pain.

Anæsthesia having been produced, the reduction was readily affected by seizing the heel with both hands, while the thumbs pushed back the two bones of the leg. The injured limb was then placed in the silicated dressing, though there was no tendency to reproduce the displacement. Result: Complete recovery.

The interesting points in the case were, (*a*) the rarity of the lesion. (Malgaigne collected but 18 cases of this kind.) (*b*) The absence of fracture described by many authors as well nigh constant. (*c*) The readiness of reduction, due to the anæsthetic and absence of fracture. (*d*) The mode of production of the injury—forced extension of the foot upon the leg and forward movement of the tibio-peroneal body.

11. *Extensive Compound Fracture of Skull and Loss of Brain Substance. Recovery.* SLOCUM. (*The Med. Record*, Dec., 1876.)

An extensive compound comminuted fracture of the skull, with escape of brain substance, and severe hæmorrhage from the middle meningeal artery, happened a lad of 15 years. He remained in a comatose condition for 15 hours; after which time co-extensive symptoms of concussion and compression were developed. Operation was made 22 hours after accident. Found fracture involved right parietal bone in its posterior inferior edge and angle, extending into occipital. Two of the largest fractured pieces, measuring 2⅝x1½ inches, were turned inward pulpifying the brain substance to a depth equal their width. The wound discharged freely of the broken brain during and after the operation. The end of the third week the hemisphere had " settled back to its normal position, followed by the scalp," leaving a depression of an inch. The mental faculties, though slow in action, were in a measure restored. The author concludes by saying: That this case argues against the policy of non-interference, and that an opening through the scalp and skull, sufficient in size to allow the entire escape of fluids, should be left. W. F L.

12. *On the Use of the Balsam of Peru.* WISS. (*Deutsche Med. Wochenschr.*, 1876, No. 48.)

In a paper read before the Berlin Medical Society, Dr. W. summed up his experience with the Peruvian balsam as follows:

The balsam, at first moment when applied to the wound, causes a slight burning, but afterward all pain ceases, even in the most severe and painful wounds.

Fresh wounds never inflamed under this treatment; and in inflamed wounds the inflammation quickly subsided.

The balsam prevented supperation, and putrid decomposition was not observed in a single case, notwithstanding the most unfavorable local and climatic conditions under which the patient had to live.

Even lacerated wounds showed a great tendency toward healing by first union. One night the doctor was summoned to attend a young man whose scalp had, by some blunt instrument, been torn into three flaps, so that the skull was laid bare to great extent. The bleeding arrested and the wound cleaned, Peruvian balsam was freely applied on the raw surfaces, which then were dressed with a simple compress. The next day the wounds were healed except a small spot in their center which also closed up in a few days, without the discharge of a drop of pus.

The anti-purulent property of the balsam induced Dr. W. to test its efficiency in cases of chronic pulmonary catarrh with a copious muco-purulent secretion. He gave the balsam in an emulsion of the yolk of eggs (ʒ j ad ℥ iv, a tablespoonful every two hours). Two elderly persons afflicted with chronic blennorrhœal bronchitis for several years, were completely and permantly restored, the one within ten days and the other within three weeks.

